# Prioritizing Zoonoses for Global Health Capacity Building—Themes from One Health Zoonotic Disease Workshops in 7 Countries, 2014–2016

**DOI:** 10.3201/eid2313.170418

**Published:** 2017-12

**Authors:** Stephanie J. Salyer, Rachel Silver, Kerri Simone, Casey Barton Behravesh

**Affiliations:** Centers for Disease Control and Prevention, Atlanta, Georgia, USA

**Keywords:** One Health, global health, zoonoses, disease prioritization, global health security, Global Health Security Agenda, International Health Regulations, IHR 2005, GHSA

## Abstract

Zoonotic diseases represent critical threats to global health security. Effective mitigation of the impact of endemic and emerging zoonotic diseases of public health importance requires multisectoral collaboration and interdisciplinary partnerships. The US Centers for Disease Control and Prevention created the One Health Zoonotic Disease Prioritization Tool to help countries identify zoonotic diseases of greatest national concern using input from representatives of human health, agriculture, environment, and wildlife sectors. We review 7 One Health Zoonotic Disease Prioritization Tool workshops conducted during 2014–2016, highlighting workshop outcomes, lessons learned, and shared themes from countries implementing this process. We also describe the tool’s ability to help countries focus One Health capacity-building efforts to appropriately prevent, detect, and respond to zoonotic disease threats.

Emerging and endemic zoonotic diseases pose a threat not only to the health of animals and humans but also to global health security. An estimated 60% of known infectious diseases and up to 75% of new or emerging infectious diseases are zoonotic in origin (*1*,*2*). Globally, infectious diseases account for 15.8% of all deaths and 43.7% of deaths in low-resource countries (*3*,*4*). It is estimated that zoonoses are responsible for 2.5 billion cases of human illness and 2.7 million human deaths worldwide each year (*5*). Emerging zoonoses are responsible for some of the most high profile and devastating epidemics (*6*–*8*); however, endemic zoonoses (*9*,*10*) may actually pose a more insidious and chronic threat to both human and animal health. As one comparison, the 2014 Ebola epidemic was responsible for 11,316 deaths and $2.2 billion in economic losses (*11*), whereas each year rabies accounts for ≈59,000 human deaths and roughly $8.6 billion in economic losses worldwide (*12*). The global impacts of emerging and endemic zoonoses on both human and animal populations make fostering collaboration between human and animal health sectors using a multisectoral, One Health approach a critical step toward improving animal and human health.

Early detection of zoonotic pathogens through enhanced laboratory capacity and surveillance at the animal–human interface is a crucial step toward controlling and preventing zoonoses (*13*–*20*) and a core capacity for implementation of the World Health Organization International Health Regulations 2005 (IHR 2005) and the Global Health Security Agenda (GHSA; https://www.ghsagenda.org/) (*21*). Rapidly detecting, responding to, and controlling public health emergencies at their source, including those caused by outbreaks of zoonotic diseases, is essential for global health security. However, in low-resource settings, capacity-building efforts should be initially focused on a few key diseases (*22*). Disease prioritization enables effective capacity building and resource allocation to increase surveillance, guide research, and improve preparedness and response protocols, further advancing global health security and the international health regulations (*23*–*25*).

To address this prioritization need, the US Centers for Disease Control and Prevention (CDC) developed the One Health Zoonotic Disease Prioritization (OHZDP) tool (*22*,*26*) as a multisectoral approach to rank a country’s zoonotic diseases using an objective, semiquantitative method. The OHZDP tool enables a country or region to bring together representatives from human, animal, and environmental health sectors to prioritize the endemic and emerging zoonoses of greatest national concern that should be jointly addressed by human, animal, and environmental health ministries using country- or region-specific criteria. Zoonotic diseases can be prioritized even in the absence of reliable prevalence data by using alternative measures for disease burden so that outcomes are provided in a timely manner, enabling country representatives to give immediate feedback, develop action plans, and capitalize on collaborations built during the prioritization process.

During 2014–2016, CDC implemented 7 OHZDP workshops. We summarize overarching themes identified from these workshops and highlight successes and lessons learned to best support additional countries in prioritizing zoonotic diseases by using this tool.

## Methods

CDC conducted OHZDP workshops using methods previously described (*22*,*26*). CDC maintains a pool of trained OHZDP workshop facilitators to conduct workshops and to train in-country facilitators to promote country ownership of the prioritization process and to leave the capacity to conduct future prioritization workshops in each country. We interviewed workshop facilitators, reviewed data from workshop materials maintained as part of our routine monitoring and evaluation activities, and reviewed available publications for information on alternate methods and outcomes. Variables collected are number and type of workshop participants by sector (voting members and observers), disease assessment criteria selected during the workshop and the resulting zoonoses rankings, and outcomes or planned next steps for multisectoral capacity building activities. Where appropriate, data for certain variables (e.g., disease ranking criteria) were standardized and combined into larger categories to look for overarching themes. Data were analyzed using Microsoft Excel (Microsoft, Redmond, WA, USA).

## Results

During 2014–2016, at countries’ request, CDC conducted OHZDP workshops in Thailand, Kenya (*27*), Ethiopia (*28*), Azerbaijan, Cameroon, South Africa, and the Democratic Republic of the Congo. All countries prioritized diseases on a national level; 4 (57.1%) workshops were specifically conducted to advance GHSA implementation in the country, but all countries had a goal to strengthen multisectoral collaboration and focus laboratory, surveillance, and prevention efforts. All workshops took place over a 2-day period, with an additional 1–2 days for training local facilitators, when requested.

All workshops used standard methods as previously described (*22*,*26*) for conducting the preworkshop activities and the in-country facilitated group work. Two countries (Kenya and Thailand) diverged from the standard methods by including more than the recommended number of voting members. Kenya placed voting members into 5 groups, then used group discussion and consensus to assign weights to the individual criteria (*27*). Thailand held 2 separate, concurrent workshops that produced 2 different outcomes; these outcomes were then combined at a separate meeting held 1 month later to develop a final list of criteria by discussion and consensus. Members were then grouped by their agencies and voted on the ranking or weight applied to each criterion before conducting a final ranking of the diseases.

### Facilitators and Participants

Fourteen CDC-trained OHZDP workshop facilitators were used for the 7 workshops and represented interdisciplinary backgrounds with expertise in zoonoses. A total of 21 in-country facilitators were trained at 5 of the 7 workshops, with an average of 4 (range 2–6) facilitators per workshop. In-country facilitators represented ministries of health (n = 8), agriculture (n = 5), environment (n = 1), and wildlife (n = 1); research institutes (n = 2); CDC in-country staff (n = 2); and other partners (n = 2). Field Epidemiology Training Program graduates were a resource for in-country facilitators in 2 workshops. Postworkshop debrief meetings and CDC facilitator interviews revealed specific lessons. For example, facilitators who held high-level positions were not available for the entire workshop because of competing priorities. In addition, it was deemed important that in-country facilitators be seen as unbiased during the facilitation process.

A total of 107 voting members participated in the 7 workshops (range 5–33), and multiple sectors were represented (Table 1). The average number of voting members per workshop was 15, but excluding 2 outlier workshops that grouped voting members (Kenya, n = 33; and Thailand, n = 22), the average was 10 (range 5–11).

**Table 1:** Sectors represented by voting members, voting members per workshop, and percentage of voting members by sector for One Health Zoonotic Disease Prioritization workshops in 7 countries, 2014–2016*

Sector and no. workshops where present	Median no. voting members/workshop (IQR)	% Total for all workshops (range)
Public health, n = 7	5 (3–6)	35.5 (16.7–50.0)
Animal health, n = 7	5 (2.5–6.5)	30.8 (16.7–50.0)
Wildlife, n = 2	5.5 (3.75–7.25)	10.3 (0–40.1)
Research institution, n = 3	3 (2–5)	10.3 (0–25.0)
Environmental health, n = 3	1 (1–2)	4.7 (0–25.0)
Local universities, n = 3	1 (1–2)	4.7 (0–25)
International partners,† n = 2	1.5 (1.25–1.75)	2.8 (0–8.3)
One Health coordinating mechanism, n = 1	1 (1–1)	0.9 (0–8.3)

*The total number of voting members for all workshops was 107. Countries: Thailand, Kenya, Ethiopia, Azerbaijan, Cameroon, South Africa, Democratic Republic of the Congo. IQR, interquartile range. †International partners were the Food and Agriculture Organization of the United Nations, International Livestock Research Institute, and the World Health Organization.

Six of workshops included observers from partner organizations or ministries. The number of observers averaged 10 (range 1–26) per workshop. Observers typically included in-country representatives from ministry partners, universities and research institutes, the World Health Organization, the Food and Agriculture Organization of the United Nations, Defense Threat Reduction Agency, the US Agency for International Development and its implementing partners, and CDC.

### Zoonotic Disease Lists

All countries provided an initial list of zoonotic diseases from the relevant ministries to the OHZDP core planning team. Many of these lists were initially created by referencing the countries’ human and animal health sector reportable disease lists. The presence of a reportable disease list did not reflect the surveillance capacity, and this variable, if selected, was assessed on-site by in-country subject matter experts. The core planning team conducted an extensive country and regionally specific literature review on the disease list. Voting members reviewed and approved the disease list on the first day of the workshop for use in the prioritization process.

Each list, on average, included 37 (range 25–43) diseases or syndromes. Zoonoses on these lists were classified as 41.4% (range 27.8%–51.3%) bacterial, 37.7% (range 28.0%–44.4%) viral, 18.3% (range 13.9%–25.0%) parasitic, 2% (range 0%–11.1%) fungal, and 0.8% (range 0%–4%) prion in nature. All lists included endemic and emerging zoonotic diseases relevant to the country or region.

All 7 initial country lists included the following bacterial zoonoses: anthrax, brucellosis, leptospirosis, plague, Q fever, salmonellosis, and zoonotic tuberculosis. All lists also included the following viral zoonoses: Crimean-Congo hemorrhagic fever; coronaviruses, including Middle East respiratory syndrome and severe acute respiratory syndrome; flaviviruses, including yellow fever and West Nile; hemorrhagic fever viruses, including Ebola and Marburg; rabies; and zoonotic influenza viruses. Six of the country lists included the following parasitic diseases: cysticercosis or taeniasis, echinococcosis, and toxoplasmosis.

### Prioritization Criteria

Six of the 7 countries selected 5 disease-ranking criteria; 1 country selected 6 criteria. All selected criteria were categorized into 7 overarching topic areas; 4 of those topics were further broken down into 2–3 more specific subtopics (Table 2). All 7 countries ranked diseases on the basis of social, economic, or environmental impact. Six of 7 countries ranked zoonotic diseases on the basis of availability of proven interventions, epidemic or pandemic potential, and severity of disease in humans; 5 ranked zoonoses on the basis of documented presence of disease in the country or region.

**Table 2:** Disease ranking criteria chosen by country during One Health Zoonotic Disease Prioritization workshops in 7 countries, 2014–2016*

Disease ranking criteria	No. countries	Average assigned weight† (range)
Economic, environmental, and/or social impact	7	0.193 (0.150–0.210)
Economic impact only	3	
Economic and/or social impact	2	
Economic, environmental, and/or social impact	2	
Availability of interventions (i.e., vaccines and/or medical treatment)	6	0.183 (0.160–0.200)
Epidemic/pandemic potential (and/or sustained transmission in humans)	6	0.202 (0.170–0.220)
Human-to-human transmission potential	5	
History of previous outbreaks	1	
Severity of disease in humans	6	0.206 (0.180–0.230)
Case-fatality rate	3	
Morbidity and/or mortality rate	3	
Presence of disease in country and/or region	5	0.200 (0.170–0.210)
Human and/or animal cases of illness reported in country and/or region‡	4	
Human or animal disease prevalence and distribution in country	1	
Laboratory capacity/diagnostic testing capacity	2	0.179 (0.160–0.198)
Existing multisectoral collaboration	2	0.183 (0.170–0.195)
Bioterrorism potential	1	0.194
Mode of transmission	1	NA

*Countries: Thailand, Kenya, Ethiopia, Azerbaijan, Cameroon, South Africa, Democratic Republic of the Congo. NA, not applicable. †Thailand was excluded from this weighting analysis since the method used in this pilot workshop differed from the standard method adopted for all future workshops.  ‡One country looked at human cases only; the other 3 looked at both human and animal cases.

When looking at the weighting, or level of importance, voting members assigned severity of disease in humans and epidemic/pandemic potential as the 2 criteria with the highest average weight. Next were documented presence of disease in the country or region, and economic, environmental, or social impact. Last, availability of proven interventions and all other remaining criteria categories were assigned the lowest weight. However, no single criterion stood out across all 7 workshops.

### Criteria Questions and Responses

Six of the 7 countries created 1 single or compound question for each selected criterion. One country created 2 separate questions for 4 of their 5 criteria, for a total of 9 questions. Voting members chose ordinal variables for all responses assigned to each criteria question. Seven (17.5%) questions had a binary response (yes/no), whereas most (82.5%) had >3 possible responses per criteria question. Regardless of the number of responses per question, all scores were normalized among criteria by using standard OHZDP tool methods (*22*).

A higher ordinal value (or score) was assigned to the responses for each question that correlated with a more severe, or negative, outcome. For example, a disease with a 50% case-fatality rate would receive a higher ordinal value than a disease with a 10% case-fatality rate. For questions that evaluated existing preventive measures, diagnostic capacity, and multisectoral collaboration, a higher ordinal score was given to responses indicating existing capacity or resources. For example, a zoonosis that could be diagnosed in the country would receive a higher score than one that could not.

### Zoonotic Disease Ranking

As a result of the tool’s ranking process in these 7 countries, 19 diseases or syndromes were ranked as prioritized diseases (Table 3). Of those, zoonotic influenza virus (n = 5), rabies (n = 5), brucellosis (n = 5), and anthrax (n = 4) were ranked by the most countries. Four of the 7 countries ranked a mix of endemic and emerging zoonoses; 2 ranked only endemic zoonoses (*27*,*28*), and 2 ranked only emerging zoonoses. Of the 4 countries that listed endemic and emerging diseases, on average, 76% (range 60%–83%) of the zoonoses on the final list were known to be endemic in the country. Six countries ranked viral, bacterial, and fungal zoonoses, and 2 countries also ranked parasitic diseases; 1 country ranked only viral diseases.

**Table 3:** Top zoonotic diseases prioritized by the One Health Zoonotic Disease Prioritization Tool for 7 countries, 2014–2016*

Zoonosis	No. countries listing disease, by rank order						Total no. countries
1	2	3	4†	5‡	6
Brucellosis (*Brucella abortus* and *B. melitensis*)		1	1§	4§			5§
Rabies	3		2				5
Zoonotic influenza			2		3		5
Anthrax	2	1	1				4
Hemorrhagic fevers (Ebola/Marburg)		1			2		3
Salmonellosis		1		2			3
Arbovirus infections (e.g., yellow fever and West Nile virus)			1			1	2
Crimean-Congo hemorrhagic fever	1			1			2
Echinococcosis		1					1
Hantavirus infection				1			1
Hendra virus infection					1		1
Leptospirosis				1			1
Monkeypox					1		1
Nipah virus infection		1					1
Q fever				1			1
Rift Valley fever					1		1
SARS					1		1
Trypanosomiasis		1					1
Zoonotic tuberculosis (*Mycobacterium bovis*)	1						1

*Countries: Thailand, Kenya, Ethiopia, Azerbaijan, Cameroon, South Africa, Democratic Republic of the Congo. SARS, severe acute respiratory syndrome. †One country had 4 diseases that shared the no. 4 ranking place. ‡One country had 4 diseases that shared the no. 5 ranking place. §One country had both *B. abortus* and *B. melitensis* on its ranked list.

### Final Prioritized List of Zoonotic Diseases

Four of the 7 counties used the original zoonoses produced by the OHZDP tool as their final prioritized list. Two countries agreed to adjust their lists to incorporate other zoonoses that the voting members felt should be in the top 5, and 1 country chose to adjust the order of the rankings to better reflect importance but retained the same zoonoses. Five countries chose a final list of 5 prioritized zoonoses, 1 country chose 6, and 1 country chose 3.

The most common zoonoses seen on the final prioritized lists remained the same as the original ranked list with the exception that rabies was selected in an additional country and brucellosis was removed in 1 country (Table 4). Five of the seven countries included both endemic and emerging zoonoses on their final prioritized lists; 69% (range 33%–83%) of these prioritized zoonoses were considered endemic to the country prioritizing the disease. Two countries prioritized only endemic zoonoses (*27*,*28*). All of the emerging zoonoses prioritized by each country were viruses. All voting members came to consensus on the final prioritized zoonoses list, modified or not. This final list was then endorsed and adopted by the participating ministries.

**Table 4:** Final combined prioritized list of zoonoses by the One Health Zoonotic Disease Prioritization Tool for 7 countries, 2014–2016*

Zoonosis	No. countries listing disease, by rank order						Total no. countries
1	2	3	4	5	6
Rabies	4		2				6
Zoonotic influenza			3		2		5
Anthrax	2	2					4
Brucellosis (*Brucella abortus and B. melitensis*)		1	2†*	2†*			4†*
Hemorrhagic fevers (Ebola/Marburg)		2		1			3
Salmonellosis		1		1			2
Zoonotic tuberculosis (*Mycobacterium bovis*)	1				1		2
Arbovirus infections (e.g., yellow fever and West Nile virus)						1	1
Crimean-Congo hemorrhagic fever				1			1
Echinococcosis					1		1
Leptospirosis				1			1
Monkeypox					1		1
Rift Valley fever					1		1
Trypanosomiasis		1					1

*Countries: Thailand, Kenya, Ethiopia, Azerbaijan, Cameroon, South Africa, Democratic Republic of the Congo. †One country had both *B. abortus* and *B. melitensis* ranked separately on the final prioritized list.

## Outcomes

Six of 7 countries planned follow-up activities as part of the workshop. Twenty postworkshop action themes were identified (Table 5). All 6 countries sought to ensure that the final prioritized list and any after-action items were approved by all participating ministries. Developing or updating and approving some type of national One Health strategy, guiding principles, or workplan was also universally identified as a desired outcome of this prioritization process. Four of the 6 countries indicated plans to use this list to establish recurring meetings, a multisectoral One Health working group or coordinating mechanisms, or both; 1 country that did not list this as an outcome already has a One Health coordination mechanism in place. The remaining action areas focused on various aspects of capacity building (Table 5).

**Table 5:** Categorized action item themes from One Health Zoonotic Disease Prioritization Workshops for 6 countries, 2014–2016*

Action item themes	Total no. workshops
Obtain ministry approval of prioritized list and activities	6
Obtain ministry support of a new or updated national plan	6
Develop a national One Health strategy, guiding principles, or work plan	5
Identify funding and technical assistance	4
Create a One Health coordinating mechanism	3
Improve data sharing across sectors	3
Establish recurring meetings	3
Develop disease-specific subcommittees	3
Strengthen the One Health workforce	3
Improve community outreach/communication	3
Improve surveillance	2
Perform a One Health capacity gap analysis	2
Link activities back to GHSA/IHR 2005	2
Improve reporting	2
Conduct research studies	2
Improve or develop laboratory capacity	1
Improve prevention and control	1
Improve outbreak response	1
Evaluate One Health impact	1
Perform the prioritization on local level	1

*Countries: Thailand, Ethiopia, Azerbaijan, Cameroon, South Africa, Democratic Republic of the Congo. Kenya was excluded because it had plan already in place before the prioritization workshop that it continued to support. GHSA, Global Health Security Agency; IHR 2005, International Health Regulations 2005.

Kenya, which did not plan postworkshop activities, had previously created a One Health strategic plan in 2012 (*29*). Their plan included many of the same capacity-building activities stated by other countries, and prevention and control activities were already under way for 4 of the 5 prioritized zoonoses. Kenya’s prioritized list validated existing activities and enabled the Zoonotic Disease Unit, the One Health coordinating mechanism for Kenya, to garner further support from the Government of Kenya to continue these efforts.

## Discussion

During 2014–2016, CDC successfully carried out 7 OHZDP workshops in Thailand, Kenya (*27*), Ethiopia (*28*), Azerbaijan, Cameroon, South Africa, and the Democratic Republic of the Congo. Several other tools and methods have been applied to prioritize zoonotic diseases (*30*–*36*), but the OHZDP process is unique in that it enables country-led decisions using a multisectoral approach to prioritize both emerging and endemic zoonotic diseases while strengthening One Health collaborations and developing action plans to build capacity for the prioritized zoonoses. In addition, the OHZDP tool can meet the needs of those working in areas where quantitative data on zoonoses are lacking. Last, the OHZDP process provides outcomes in a timely manner so that participants may give immediate feedback and capitalize on One Health collaborations built during the prioritization process.

We have found key successes and lessons learned through the review of these workshops. First, successful outcomes are dependent on trust, transparency, equal representation, and consensus from all relevant sectors participating in the prioritization process and approving the final prioritized list of zoonoses. The CDC-trained OHZDP workshop facilitators not only conduct workshops but also train in-country facilitators to promote country ownership of the process and to build in-country capacity to conduct future workshops. Trained facilitators ensure that the prioritization process is standardized and conducted effectively. We found that using an interdisciplinary team of trained facilitators who remained neutral, unbiased, and did not focus on their specific sector, affiliation, or area of expertise enabled voting members’ voices to be heard and recognized. Our review found that most voting members were from the human (35.5%) and animal (30.8%) health sectors, but additional sectors were represented where available, ensuring the multisector nature of this process.

To accommodate a larger number of voting participants, methods were modified in 2 workshops. However, because these methods have not been rigorously tested, it is still advised that future workshops maintain the recommended number of participants (8 to 12) to enable more focused discussion during and timely results from the 2-day workshop.

Funding partner advocacy and support of the process and future activities is a potential benefit of observer participation. However, care is needed to ensure that the number of observers in their role as advisors and participants during discussions do not overwhelm or influence the process. Keeping to the recommended 10–15 total observers (*26*) is needed so that voting members can focus on the workshop process. We recommend having an overview summary at the end of the workshop that is open to a larger group of higher level in-country representatives and other partners to share the workshop outcomes in a timely way.

The OHZDP tool was designed to accommodate diversity in location (i.e., globally) and scale (i.e., local, national, regional) into the prioritization process so participants can select criteria relevant to their needs. We found that most countries were interested in selecting criteria that targeted zoonoses known to be present in country with the following attributions: high illness and death rates in humans; pandemic potential; availability of proven interventions; and economy, environment, or societal impact. Most prioritized zoonoses were endemic diseases, illustrating that countries wanted to first focus their limited resources on diseases for which they could successfully implement enhanced diagnostic capacity, surveillance, and proven interventions.

Common priority action items identified in these workshops are highly relevant to advancing global health security, including improving data sharing between ministries, improving communication to the public, strengthening the One Health workforce, developing disease-specific subcommittees, and increasing general surveillance and outbreak response capacity. Such activities will enhance the capacity of countries to rapidly detect, respond to, and contain public health emergencies, including outbreaks of zoonotic diseases, at their source and thereby ensure global health security. Most countries with identified priority action items planned to use this list to solicit or engage funding partners, which highlights countries taking ownership of the prioritization process, and recognizing and advocating for support around their country-specific priorities. Six countries made sure that the prioritized list and any after-action items were approved by all participating ministries and that a national One Health strategy or multisectoral coordination mechanism was established if it had not been already. By forming or hosting these prioritization workshops with a ministerial One Health coordinating committee, these after-action plans are more readily taken up.

Four of the 7 countries conducted this activity to meet Joint External Evaluation and GHSA zoonotic disease prioritization and collaboration goals. The next step is that these countries then build these plans into their existing activities. These countries are supported by global health partners to help meet these goals.

As part of the continual improvement process for the OHZDP tool, we are employing postworkshop evaluations, in addition to continuing the postworkshop debriefs and facilitator interviews to ensure that these workshop continue to have successful outcomes. Moving forward, lessons learned from OHZDP workshops conducted during 2014–2016 will be applied to standardize and enhance the prioritization process in the future.

All 7 prioritizations were conducted during or in the wake of the 2014 West Africa Ebola outbreak (*11*). This event likely influenced the outcome for 1 country that prioritized Ebola despite the disease not being endemic or a likely risk in the country or region. Periodically repeating this prioritization process could help eliminate bias from current events, as well as aid in reevaluating if currently prioritized diseases still pose a public health threat, if sufficient capacity has been built, and if newly emerging diseases or other zoonoses need to be considered.

In summary, the GHSA uses a One Health multisectoral approach to strengthen the capacity at the global and national levels to prevent, detect, and respond to human and animal infectious disease threats, whether naturally occurring or accidentally or deliberately spread, that threaten global health security. Both endemic and emerging zoonotic diseases are recognized as being critical for global health security and related efforts. The OHZDP tool aids the GHSA mission by helping countries and regions prioritize their zoonotic diseases of greatest national concern and focusing GHSA capacity-building efforts on improving laboratory capacity, surveillance, outbreak response, and prevention activities on a few key zoonoses at first. The OHZDP process also supports progress toward the Joint External Evaluation, specifically for the zoonotic disease indicators, on having national laboratory, surveillance, and joint outbreak response plans and strategies in place for priority endemic/emerging zoonotic diseases with evidence of a multisectoral, coordinated approach. A multisectoral zoonotic disease prioritization with equal engagement from all sectors active in zoonotic disease work is one of the most cost-effective ways a country, especially one with limited resources, can begin using a One Health approach to prevent, detect, and respond to public health threats. By building these capacities and strengthening One Health partnerships for prioritized diseases, a country will not only more effectively address existing diseases but also have the systems in place to be better prepared to detect and respond to new and emerging diseases that may occur and become a threat to global health security.
